# Socioeconomic inequalities in physiological risk biomarkers and the role of lifestyles among Russians aged 35-69 years

**DOI:** 10.1186/s12939-022-01650-3

**Published:** 2022-04-15

**Authors:** Sergi Trias-Llimós, Sarah Cook, Anne Elise Eggen, Alexander V. Kudryavtsev, Sofia Malyutina, Vladimir M. Shkolnikov, David A. Leon

**Affiliations:** 1 Department of Non-communicable Disease Epidemiology, Faculty of Epidemiology and Population Health, London School of Hygiene & Tropical Medicine, London, UK; 2 Centre d’Estudis Demogràfics, Centres de Recerca de Catalunya (CERCA), Carrer de Ca n’Altayó, Edifici E2, Universitat Autònoma de Barcelona, 08193 Bellaterra, Spain; 3 National Heart and Lung Institute, Imperial College London, London, UK; 4 Department of Community Medicine, Faculty of Health Sciences, UiT The Arctic University of Norway, Tromsø, Norway; 5 Central Scientific Research Laboratory, Northern State Medical University, Arkhangelsk, Russia; 6 Research Institute of Internal and Preventive Medicine - Branch of IC&G SB RAS, Novosibirsk, Russia; 7 Novosibirsk State Medical University, Ministry of Health of Russia, Novosibirsk, Russia; 8 Laboratory for Demographic Data, Max Planck Institute for Demographic Research, Rostock, Germany; 9 International Laboratory for Population and Health, National Research University Higher School of Economics, Moscow, Russia

**Keywords:** Cardiovascular risk, Health inequities, Health behaviours, Russia, Eastern Europe

## Abstract

**Background:**

Socioeconomic inequalities in cardiovascular (CVD) health outcomes are well documented. While Russia has one of the highest levels of CVD mortality in the world, the literature on contemporary socio-economic inequalities in biomarker CVD risk factors is sparse. This paper aims to assess the extent and the direction of SEP inequalities in established physiological CVD risk biomarkers, and to explore the role of lifestyle factors in explaining SEP inequalities in physiological CVD risk biomarkers.

**Methods:**

We used cross-sectional data from a general population-based survey of Russians aged 35-69 years living in two cities (*n* = 4540, Know Your Heart study 2015-18). Logistic models were used to assess the associations between raised physiological risk biomarkers levels (blood pressure levels, cholesterol levels, triglycerides, HbA1C, and C-reactive protein) and socioeconomic position (SEP) (education and household financial constraints) adjusting for age, obesity, smoking, alcohol and health-care seeking behavior.

**Results:**

High education was negatively associated with a raised risk of blood pressure (systolic and diastolic) and C-reactive protein for both men and women. High education was positively associated with total cholesterol, with higher HDL levels among women, and with low triglycerides and HbA1c levels among men. For the remaining risk biomarkers, we found little statistical support for SEP inequalities. Adjustment for lifestyle factors, and particularly BMI and waist-hip ratio, led to a reduction in the observed SEP inequalities in raised biomarkers risk levels, especially among women. High financial constraints were weakly associated with high risk biomarkers levels, except for strong evidence for an association with C-reactive protein (men).

**Conclusions:**

Notable differences in risk biomarkers inequalities were observed according to the SEP measure employed. Clear educational inequalities in raised physiological risk biomarkers levels, particularly in blood pressure and C-reactive protein were seen in Russia and are partly explained by lifestyle factors, particularly obesity among women. These findings provide evidence-based information on the need for tackling health inequalities in the Russian population, which may help to further contribute to CVD mortality decline.

**Supplementary Information:**

The online version contains supplementary material available at 10.1186/s12939-022-01650-3.

## Background

Cardiovascular disease (CVD) is the largest single cause of death worldwide [1]. CVD mortality is particularly high among middle aged individuals in Eastern Europe, and Russia in particular [1, 2]. For example, the age-standardized rates of 1212 per 100,000 among Russian males and 756 per 100,000 among Russian females in 2014 contrasts with the equivalent rate in the neighbouring country of Norway of 287 and 209 per 100,000 observed among males and females, respectively (in 2016) [1]. The potential contribution of lifestyle and socioeconomic factors to CVD health has been described in several reviews [3–7]. The elevated Russian CVD mortality is driven by multiple factors including individual-level determinants such as lifestyle and established cardio-metabolic factors [8], and levels of investment in the health sector, particularly cardiovascular treatment technology and staff training [9, 10].

The existence of potentially large socioeconomic inequalities and high mortality in disadvantaged groups may also contribute to overall levels of CVD mortality [11, 12]. Socioeconomic position inequalities (SEP) refers to the social and economic factors that influence what positions individuals or groups hold within the structure of a society [13]. Indeed, SEP inequalities in CVD incidence and mortality are important in Europe across a wide range of socioeconomic position measures [14, 15]. Eastern European countries, includin Russia, are known for their large socioeconomic inequalities in all-cause and cardiovascular mortality [9, 11, 12, 15–20]. However, less is known about socioeconomic differences in biomarkers and physiological pathways underlying the large inequalities in mortality and health in Russia.

There is consistent evidence that riskier lifestyles and risk factor profiles exist in Russia in comparison with Western European countries, at least in high alcohol use [21], smoking [22] and high blood pressure levels [23]. Lifestyle factors, such as smoking or alcohol, and established CVD risk factors, such as raised blood pressure and cholesterol levels, predict CVD health outcomes, and provide potential pathways along with socioeconomic circumstances impact on CVD. For example, results derived from the HAPIEE study found a clear educational gradient in CVD mortality in Eastern Europe, which diminished after adjusting for established CVD risk factors [24]. A small number of other studies have focused on links between socioeconomic inequalities and health outcomes in Russia. Several of these have explored the mediating role of established cardiometabolic risk factors using data from the Lipid Research Clinics (LRC) and MONICA studies aged 35 to 64 years, and they found that established risk factors can potentially explain 20 to 30% of the educational differences in the risk of CVD mortality [18, 20].

More recent studies have focused on older ages using data from the Survey on Stress, Ageing and Health in Russia (SAHR) collected in 2006-2009 (1800 Moscow residents aged 55 and older, 68 years on average). This found clear educational gradients in established cardiovascular factors and markers of inflammation [25], in which biomarkers were found to explain 19 and 36% of educational differences in general health and physical function, respectively. A further analysis of SAHR data looked at educational impacts on all-cause and CVD mortality [19]. This confirmed the importance of established risk factors and markers of inflammation in mediating the relationship between level of education and death. However, despite these previous studies, there has not been any focussed attempt to look at SEP inequalities and physiological CVD risk factors with individual-level lifestyle factors in Russia.

This paper aims to contribute to the understanding of SEP inequalities in CVD in Russia by addressing the following objectives: 1) To assess the extent and the direction of SEP inequalities in established physiological CVD risk biomarkers, and 2) to explore the role of lifestyle factors in explaining SEP inequalities in physiological CVD risk biomarkers. We hypothesize that SEP inequalities in risk biomarkers exist, and we will test the extent to which this is true for the biomarkers analyzed, using two alternative measures of SEP, and the extent to which behavioural factors mitigate theses associations.

## Methods

To fulfill our objectives, we use two measures of SEP: highest educational attainment and self-perceived household financial constraints. We used data from two large provincial Russian cities. Our focus on working age groups is consistent with the common finding that SEP differentials for CVD are particularly pronounced at this younger age.

### Data

We used data from the population-based cross-sectional survey Know Your Heart (KYH) study carried out in men and women aged 35-69 years in the cities of Arkhangelsk and Novosibirsk (Russia) between 2015 and 2018 [26]. The KYH Study is one of the major components of The International Project on Cardiovascular Disease in Russia (IPCDR), which aimed to provide a more comprehensive understanding of the reasons for the extremely high cardiovascular mortality in Russia, and which provides one of the most recent and detailed health survey and biomarker information for Russian populations. KYH consisted of a baseline interview completed in participants’ homes by a trained interviewer and a health check at a polyclinic (a health centre providing outpatient care) completed by medical professionals (both doctors and nurses). Detailed standard operating procedures were followed to ensure standard collection of the physiological parameters in each site. Review of the quality of data from both sites was conducted centrally monthly throughout the study and feedback provided to the clinical sites. Within each of the two cities, four districts were selected purposefully to represent a range of socio-demographic and mortality levels in each city. The sampling frame was provided by the regional health insurance funds and included addresses of all residents having compulsory health insurance, supplemented by information on the age and sex of occupants at individual addresses. Overall, 4560 individuals completed the interview and took part in the health check: a response rate of 51% out of the total number of those invited. Further details concerning response rates, and organization of interviewing and medical examination can be found in the KYH descriptive study by Cook et al. [26].

Dependent variables included in this study were derived by dichotomising levels of physiological CVD biomarkers. The risk biomarkers cut off points were those used in a previous SAHR study conducted in Moscow [25], except for LDL which was not included in SAHR: systolic blood pressure (SBP, cut off ≥140 mmHg), diastolic blood pressure (DBP, cut off ≥90 mmHg), total cholesterol (cut off ≥6.216 mmol/L), LDL cholesterol (cut off ≥3.8 mmol/L corresponding ot), HDL cholesterol (cut off < 1.036 mmol/L), and triglycerides (cut off ≥2.26 mmol/L), glycated hemoglobin (HbA1C, cut off > 6.5%), and C-reactive protein (CRP, cut off > 3 mg/L). These risk biomarkers are all associated with higher mortality risks, and they were selected based on the robustness and availability in the KYH. Details on the KYH sample, including the methods behind the risk biomarkers measurements, can be found elsewhere [26].

We conducted parallel analyses using two alternate measures of self-reported SEP: educational attainment (education) and household financial constraints (financial constraints). Education was dichotomized as high SEP (higher or incomplete higher education vs lower SEP (secondary or lower education) [27]. Financial constraints were dichotomized as follows: high SEP for those able to buy non-essential products (no or minimal constraint of not being able to buy large domestic appliances), and lower SEP for those with constrains to buy essential products (constrained in buying food or clothes). We acknowledge that the dichotomized SEP variables represent a limitation in terms of assessing a full spectrum of educational inequalities.

We treated age (5-year age groups) and city (Arkhangelsk or Novosibirsk) as a priori potential confounders. However, we also included in models lifestyle related-factors to see how far these might drive any observed inequalities in the CVD biomarkers: smoking behaviour (never, former, up to 10 cigarettes per day, 10-20 cigarettes per day and over 20 cigarettes per day) [28], alcohol use (non-drinkers, and two drinking categories using the Alcohol Use Disorders Identification Test (AUDIT) score [29] cut point of ≥8 to indicate hazardous or harmful drinking [30, 31]); physical activity (floors of stairs climbed per day over the last 12 months) [32, 33]; body mass index (BMI, in kg/m^2^: < 25, > = 25 and < 30, and > =30); waist-hip ratio (in cm, dichotomous, cutoff 0.85 for women and 0.90 for men) [34]; one item on diet from the Dietary Quality score (fruit intake) [35]. In addition, we adjusted for medications, again to see how far these factors might drive the observed inequalities in the CVD biomarkers (blood pressure lowering medication (Anatomical Therapeutic Chemical Classification System codes [36] C02-C03, C07-C09), lipid lowering medication (statins C10) and blood sugar lowering medication (A10A (insulin), A10B (oral blood sugar lowering drugs)); visits to the doctor over the last 12 months; and self-reported familiarity with arterial hypertension (high blood pressure).

We used complete case data for each (physiological) risk biomarker. Individuals with missing values in any of the variables of interest were excluded from the analysis (410 (9.0%) blood pressure, 140 lipids (3.1%), 195 HbA1c (4.3%), and 84 CRP (1.9%)).

To explore whether our findings were influenced by the individuals with pre-existing diseases, we perform a sensitivity analysis by excluding individuals who reported having had a myocardial infarction or stroke and or had angina on the Rose Angina Questionnaire (short version) [18] (see Table S1).

### Analytical approach

All analyses were stratified by gender. We used direct age-standardization to estimate prevalences of high-risk profiles by SEP (including 95%CI and *p*-values of the difference between educational groups) using the Russian population from 2014 as the standard from the Human Mortality Database (HMD).

Logistic regression models were run to assess the associations between SEP measures (level of education and financial constraints) and physiological risk biomarkers (prevalence of raised biomarker levels). In both sets of models we adjusted for age, city (Model 1), and a set of additional variables. Model 2 included the lifestyle variables smoking, alcohol (AUDIT-8), diet (fruit intake), and physical activity (floors of stairs climbed). Model 3 included the variables related to overweight and obesity: BMI and waist-hip ratio. Model 4 included all variables included in previous models, whereas in model 5 we additionally adjusted for risk factor specific medication use. Finally, for blood pressure, an additional model was added to account for health literacy (using blood pressure knowledge as a proxy) although the results are not shown as this had not effect. A sensitivity analysis using continuous risk factor biomarkers as dependent variables and linear regression models is included in Supplementary Material.

## Results

Our sample consisted of 1901 men and 2637 women. For both men and women around 40% were classified as having high education, whereas notable gender differences were observed when considering financial constraints as SEP. Among men, 34% were classified as of low SEP based on financial constraints, whereas among women this figure was much higher at 72%. Table 1 provides the descriptive characteristics of our sample.

**Table 1:** Descriptive characteristics of the sample, Know Your Heart, ages 35-69

		Men (*n* = 1901)		Women (2637)	
Variable	Categories	n	%	n	%
City	Arkhangelsk	989	52%	1389	53%
	Novosibirsk	912	48%	1248	47%
Age	35-39	160	8%	236	9%
	40-44	225	12%	350	13%
	45-49	255	13%	349	13%
	50-54	280	15%	380	14%
	55-59	291	15%	411	16%
	60-64	344	18%	425	16%
	65-69	346	18%	486	18%
Education	Higher (high SEP)	771	41%	1102	42%
	Lower (low SEP)	1130	59%	1535	58%
Financial constraints	Constrained (low SEP)	644	34%	1893	72%
	Unconstrained (high SEP)	1212	64%	707	27%
	Missing	45	2%	37	1%
AUDIT 8	Non-drinker	284	15%	341	13%
	Lower than 8	1156	61%	2234	85%
	Equal or higher than 8	448	24%	52	2%
	Missing	13	1%	10	0%
Smoking	Never	523	28%	1807	69%
	Former	677	36%	399	15%
	Up to 10	140	7%	229	9%
	More than 10, up to 20	424	22%	175	7%
	More than 20	128	7%	25	1%
	Missing	9	0%	2	0%
BMI kg/m^2^	Normal (< 25.0)	554	29%	819	31%
	Overweight (25.0-29.9)	839	44%	848	32%
	Obesity (30.0+)	501	26%	959	36%
	Missing	7	0%	11	0%
Waist-hip quartile	Low	470	25%	1452	55%
	High	1427	75%	1183	45%
	Missing	4	0%	2	0%
Physical activity: stairs (floors per day)	Mean	8.12		6.15	
Diet (fruit intake)	None	149	8%	99	4%
	1-2 per week	446	23%	347	13%
	3-4 per week	382	20%	410	16%
	5-6 per week	111	6%	208	8%
	1-2 per day	685	36%	1244	47%
	3-4 per day	93	5%	241	9%
	5-6 per day	21	1%	56	2%
	More than 6 per day	12	1%	31	1%
	Missing	2	0%	1	0%
Knowledge about Blood Pressure	Not at all	162	9%	113	4%
	I have only heard the term before	236	12%	169	6%
	I know a little about it	631	33%	854	32%
	I am very familiar with it	870	46%	1500	57%
	Missing	2	0%	1	0%
Blood pressure lowering medication	Yes	646	34%	1060	40%
	No	1255	66%	1577	60%

### Age-standardized risk factor means

Age-standardized prevalence of raised physiological risk biomarkers by educational attainment suggest men and women without higher education have a higher prevalence of high-risk blood pressure and CRP levels, and lower prevalence of raised total cholesterol levels among women as compared to their counterparts with higher education (Table 2). Men with financial constraints had a higher prevalence of raised triglycerides and CRP compared with those free of financial constraints, while the results for women and for the other physiological risk biomarkers did not suggest the presence of inequalities according to financial constraints.

**Table 2:** Age-standardized prevalence (in %) of raised risk physiological CVD biomarkers* by education** and household financial constraints in the Know Your Heart Study

		Men		
		Low SEP	High SEP	*p*-value
Education	SBP	38.1 (35.0-41.2)	31.9 (28.4-35.3)	**0.018**
	DBP	35.3 (32.2-38.4)	28.8 (25.4-32.3)	**0.013**
	HDL	43.1 (39.8-46.3)	45.0 (41.4-48.7)	0.081
	LDL	19.2 (16.6-21.7)	16.7 (13.9-19.5)	0.527
	TCHOL	21.9 (19.3-24.5)	21.6 (18.6-24.6)	0.994
	Triglycerides	19.5 (16.9-22.1)	18.7 (15.8-22.5)	0.803
	HbA1C	4.9 (3.7-6.2)	3.9 (2.6-5.1)	0.483
	CRP	30.0 (27.0-32.9)	24.5 (21.3-27.6)	**0.020**
Financial constraints	SBP	37.1 (34.1-40.0)	34.5 (30.7-38.2)	0.433
	DBP	32.3 (29.5-37.1)	33.3 (29.5-37.1)	0.754
	HDL	44.0 (40.9-47.1)	44.7 (40.7-48.7)	0.340
	LDL	18.8 (16.4-21.3)	17.5 (14.4-20.6)	0.676
	TCHOL	22.1 (19.5-24.6)	21.4 (18.2-24.6)	0.953
	Triglycerides	21.2 (18.6-23.8)	15.5 (12.6-18.3)	**0.015**
	HbA1C	4.8 (3.6-6.0)	3.8 (2.4-5.2)	0.223
	CRP	30.5 (27.7-33.3)	23.4 (20.0-26.8)	**< 0.001**
		Women		
		Low SEP	High SEP	*p*-value
Education	SBP	22.9 (20.8-25.0)	18.7 (16.3-21.0)	**0.004**
	DBP	19.8 (17.6-22.0)	14.6 (12.4-16.9)	**< 0.001**
	HDL	41.0 (38.4-43.5)	42.6 (39.7-45.4)	0.020
	LDL	5.7 (4.4-6.9)	3.6 (2.4-4.7)	0.555
	TCHOL	23.5 (21.4-25.6)	27.6 (25.0-30.2)	**0.021**
	Triglycerides	13.3 (11.5-15.1)	11.4 (9.4-13.3)	0.214
	HbA1C	4.5 (3.5-5.5)	3.9 (2.8-5.1)	0.592
	CRP	33.9 (31.2-36.5)	25.6 (22.9-28.2)	**< 0.001**
Financial constraints	SBP	21.2 (19.4-23.0)	19.1 (16.2-22.0)	0.201
	DBP	17.7 (15.9-19.6)	16.6 (13.8-19.5)	0.448
	HDL	41.9 (39.6-44.2)	41.6 (38.2-45.1)	0.198
	LDL	5.0 (4.0-6.1)	3.7 (2.3-5.1)	0.981
	TCHOL	25.3 (23.4-27.3)	25.1 (22.0-28.2)	0.948
	Triglycerides	12.7 (11.2-14.3)	12.2 (9.8-14.6)	0.922
	HbA1C	4.3 (3.4-5.2)	3.9 (2.6-5.3)	0.930
	CRP	31.2 (29.0-33.4)	28.0 (24.6-31.4)	0.098

* SBP stands for systolic blood pressure ≥ 140 mmHg, DBP for diastolic blood pressure ≥ 90 mmHg, HDL for high density lipoprotein < 1.036 mmol/L, LDL for low density lipoprotein ≥3.8 mmol/L, TCHOL for total cholesterol in blood ≥6.216 mmol/L, HbA1C for glycated haemoglobin > 6.5%, and CRP for C-reactive protein > 3 mg/L

** Education was categorized in: high SEP (higher or incomplete higher education), and lower SEP (secondary or lower education); and financial constraints were categorized in: unconstained or high SEP (self-perceived not to have any financial constraint or only constrained to by large domestic appliances), and constrained or low SEP (constrained in buying food or clothes)

### Regression models

High level of education was negatively associated with raised levels of blood pressure and CRP for both men and women, as well as for HDL for women (Tables 3 and 4). For example, the odds ratios (OR) of high systolic blood pressure were 0.78 (95%CI; 0.64-0.96) among men and 0.73 (0.60-0.90) among women, while for high CRP the corresponding values were 0.78 (0.63-0.96) among men and 0.66 (0.55-0.79) among women. High education was associated with raised total cholesterol levels for women. Financial constraints were found to have weak associations with risk biomarkers prevalence, Among men, the associations between raised blood pressure prevalence and education persisted after adjusting for all lifestyle-related variables and blood pressure lowering medication (e.g. OR for SBP in model 2 was 0.76 (0.62-0.94)) (Tables 3 and 4). There was only weak evidence of any associations of education with HDL, LDL, triglycerides and HbA1c among men regardless of model. Finally, the associations between high-risk CRP levels and education weakened after adjusting for lifestyles (model 2, OR: 0.91 (0.73-1.14), which is explained by smoking (model 1b, OR: 0.92 (0.74-1.14), results not shown in table). BMI and waist-hip ratio did not explain the associations between high-risk CRP levels and education (model 3, OR: 0.77 (0.62-0.95). Results for household financial constraints suggested weak SEP associations except for CRP.

**Table 3:** Odds ratios for raised risk physiological CVD biomarkers* by education and household financial constraints, men (*n* = 1901)

Men		Model 1: Age + city	Model 2: Model 1 + smoking + alcohol (AUDIT8) + fruit + stairs	Model 3: Model 1 + BMI + waist-hip	Model 4: Models 2 + 3	Model 5: Model 4 + medication
Education (ref. Low)	SBP	**0.784 (0.638, 0.962)**	**0.774 (0.625, 0.958)**	**0.793 (0.645, 0.975)**	**0.784 (0.632, 0.971)**	**0.778 (0.628, 0.965)**
	DBP	**0.776 (0.630, 0.955)**	**0.748 (0.602, 0.929)**	**0.779 (0.631, 0.963)**	**0.758 (0.608, 0.945)**	**0.756 (0.607, 0.943)**
	HDL	0.803 (0.628, 1.026)	0.870 (0.672, 1.126)	0.801 (0.623, 1.029)	0.879 (0.676, 1.144)	0.876 (0.673, 1.139)
	LDL	1.041 (0.862, 1.258)	1.011 (0.830, 1.232)	1.043 (0.861, 1.263)	1.028 (0.842, 1.256)	1.031 (0.843, 1.261)
	TCHOL	0.964 (0.770, 1.208)	0.927 (0.733, 1.174)	0.955 (0.761, 1.198)	0.926 (0.731, 1.174)	0.926 (0.730, 1.174)
	Triglycerides	0.951 (0.749, 1.207)	0.990 (0.771, 1.271)	0.948 (0.739, 1.216)	1.016 (0.784, 1.318)	1.014 (0.782, 1.315)
	HbA1C	0.846 (0.553, 1.293)	0.902 (0.580, 1.404)	0.825 (0.534, 1.276)	0.962 (0.611, 1.516)	0.954 (0.603, 1.511)
	CRP	**0.778 (0.631, 0.959)**	0.912 (0.731, 1.139)	**0.768 (0.621, 0.949)**	0.911 (0.727, 1.142)	0.908 (0.724, 1.138)
Financial constraints (ref. Low)	SBP	0.910 (0.735, 1.126)	0.935 (0.750, 1.165)	0.930 (0.750, 1.154)	0.957 (0.766, 1.195)	0.971 (0.776, 1.214)
	DBP	1.007 (0.814, 1.248)	1.034 (0.827, 1.292)	1.021 (0.820, 1.269)	1.056 (0.841, 1.326)	1.062 (0.845, 1.333)
	HDL	0.885 (0.685, 1.143)	0.923 (0.709, 1.203)	0.873 (0.672, 1.134)	0.932 (0.711, 1.223)	0.953 (0.726, 1.251)
	LDL	1.091 (0.895, 1.330)	1.078 (0.879, 1.322)	1.069 (0.873, 1.307)	1.060 (0.861, 1.305)	1.035 (0.839, 1.275)
	TCHOL	1.082 (0.855, 1.370)	1.079 (0.844, 1.378)	1.060 (0.835, 1.346)	1.050 (0.820, 1.345)	1.037 (0.809, 1.329)
	Triglycerides	0.754 (0.582, 0.977)	0.726 (0.555, 0.950)	0.738 (0.563, 0.967)	0.735 (0.556, 0.971)	0.748 (0.566, 0.990)
	HbA1C	0.773 (0.487, 1.229)	0.812 (0.503, 1.310)	0.757 (0.471, 1.217)	0.835 (0.511, 1.366)	0.900 (0.547, 1.479)
	CRP	**0.657 (0.526, 0.822)**	**0.752 (0.596, 0.950)**	**0.658 (0.525, 0.826)**	**0.760 (0.599, 0.964)**	**0.773 (0.609, 0.981)**

* SBP stands for systolic blood pressure ≥ 140 mmHg, DBP for diastolic blood pressure ≥ 90 mmHg, HDL for high density lipoprotein < 1.036 mmol/L, LDL for low density lipoprotein ≥3.8 mmol/L, TCHOL for total cholesterol in blood ≥6.216 mmol/L, HbA1C for glycated haemoglobin > 6.5%, and CRP for C-reactive protein > 3 mg/L

**Table 4:** Odds ratios for raised risk physiological CVD biomarkers* by education and household financial constraints, women (*n* = 2637)

Women		Model 1: Age + city	Model 2: Model 1 + smoking + alcohol (AUDIT8) + fruit + stairs	Model 3: Model 1 + BMI + waist-hip	Model 4: Models 2 + 3	Model 5: Model 4 + medication
Education (ref. Low)	SBP	**0.733 (0.595, 0.903)**	**0.762 (0.615, 0.944)**	**0.790 (0.638, 0.977)**	0.823 (0.661, 1.023)	0.853 (0.684, 1.064)
	DBP	**0.678 (0.542, 0.848)**	**0.730 (0.580, 0.919)**	**0.757 (0.602, 0.951)**	0.806 (0.637, 1.020)	0.826 (0.652, 1.046)
	HDL	**0.623 (0.419, 0.925)**	0.738 (0.492, 1.107)	0.749 (0.498, 1.125)	0.892 (0.586, 1.357)	0.896 (0.589, 1.363)
	LDL	1.035 (0.874, 1.226)	1.038 (0.873, 1.234)	1.082 (0.912, 1.284)	1.080 (0.906, 1.287)	1.071 (0.898, 1.276)
	TCHOL	**1.213 (1.006, 1.463)**	**1.218 (1.005, 1.475)**	**1.231 (1.019, 1.487)**	**1.231 (1.014, 1.493)**	**1.221 (1.006, 1.482)**
	Triglycerides	0.851 (0.667, 1.087)	0.867 (0.675, 1.114)	1.003 (0.778, 1.293)	1.013 (0.781, 1.313)	1.032 (0.795, 1.339)
	HbA1C	0.893 (0.609, 1.307)	0.900 (0.610, 1.327)	1.084 (0.730, 1.610)	1.066 (0.712, 1.597)	1.091 (0.727, 1.635)
	CRP	**0.657 (0.550, 0.785)**	**0.680 (0.566, 0.816)**	**0.779 (0.643, 0.945)**	**0.798 (0.655, 0.972)**	**0.810 (0.665, 0.988)**
Financial constraints (ref. Low)	SBP	0.857 (0.679, 1.083)	0.902 (0.710, 1.145)	0.886 (0.699, 1.123)	0.932 (0.731, 1.187)	0.966 (0.756, 1.235)
	DBP	0.885 (0.694, 1.129)	0.939 (0.732, 1.204)	0.926 (0.723, 1.186)	0.974 (0.755, 1.255)	0.997 (0.773, 1.287)
	HDL	0.752 (0.483, 1.171)	0.859 (0.545, 1.355)	0.821 (0.524, 1.287)	0.957 (0.601, 1.525)	0.968 (0.607, 1.544)
	LDL	1.034 (0.858, 1.246)	1.042 (0.862, 1.259)	1.043 (0.865, 1.258)	1.047 (0.866, 1.267)	1.038 (0.858, 1.257)
	TCHOL	1.054 (0.855, 1.299)	1.040 (0.841, 1.287)	1.055 (0.856, 1.301)	1.039 (0.839, 1.285)	1.028 (0.831, 1.273)
	Triglycerides	1.001 (0.766, 1.308)	0.999 (0.760, 1.313)	1.078 (0.818, 1.420)	1.065 (0.803, 1.411)	1.091 (0.822, 1.448)
	HbA1C	0.998 (0.650, 1.533)	1.023 (0.660, 1.585)	1.051 (0.677, 1.631)	1.069 (0.681, 1.678)	1.094 (0.695, 1.722)
	CRP	0.861 (0.708, 1.048)	0.888 (0.727, 1.085)	0.918 (0.742, 1.136)	0.946 (0.762, 1.174)	0.964 (0.776, 1.198)

* SBP stands for systolic blood pressure ≥ 140 mmHg, DBP for diastolic blood pressure ≥ 90 mmHg, HDL for high density lipoprotein < 1.036 mmol/L, LDL for low density lipoprotein ≥3.8 mmol/L, TCHOL for total cholesterol in blood ≥6.216 mmol/L, HbA1C for glycated haemoglobin > 6.5%, and CRP for C-reactive protein > 3 mg/L

Among women, there were negative associations between higher education and raised blood pressure. These were attenuated after adjusting for lifestyles, BMI and waist-hip ratio (model 4, OR for SBP: 0.82 (0.66-1.02); OR for DBP: 0.81 (0.64-1.02)). Finally, the associations between CRP and education weakened after adjusting for obesity-related measures. Results for household financial constraints suggested SEP differences in high-risk CRP levels (model 1, OR: 0.66 (0.53-0.82), and the OR with statistical evidence for a difference in all models.

Sensitivity analyses for continuous risk factor measures derived from linear regression models were broadly consistent with our main analyses based on binary outcomes with lower mean levels of SBP among the higher educated group in both men (beta: − 2.38 (− 4.20, − 0.57) and women (beta: − 3.11 (− 4.61, − 1.62) (Table S2). Similarly, inequalities were observed in DBP. These inequalities persisted in the multivariable models, except for DBP levels among women, which declined after adjusting for lifestyles and obesity-related measures. However, in contrast to that observed in the main results, higher educated men had lower levels of HbA1c, and these inequalities weakened after adjusting for major lifestyles factors such as smoking and alcohol (model 2). The significance of CRP levels was similar to the one presented in the main results. Finally, higher educated women presented higher HDL levels and lower HbA1c. In both cases, these differences weakened after adjusting for obesity-related measures (model 3). Similar to the findings for the high-risk factor profiles, there was a lack of evidence supporting clear inequalities according to the financial status.

## Discussion

This study has found higher education to be associated with a reduced probability of raised blood pressure (SBP and DBP) and CRP for both men and women, with raised HDL levels and lower total cholesterol levels for women. For the remaining physiological CVD risk biomarkers analyzed, we found little statistical evidence of SEP inequalities. High financial constraints were weakly associated with high risk biomarkers levels, except for strong evidence for higher levels of CRP in men. Therefore, our initial hypothesis on the existence of SEP inequalities is thus only partly confirmed. Lifestyle factors appear to be the driver of some of these observed SEP inequalities in risk factor profiles.

### Explanation of results

In this study we have used two different measure of SEP: education and household financial constraints. Each of these measures represent related but different SEP dimensions [13] and have been shown to be differently related to CVD health [37]. Education is usually completed by early adulthood and therefore is an indicator of SEP that is unlikely to change in response to ill-health later in life [38]. Recent evidence from a Mendelian randomization study provided evidence of a causative role of education in CVD risks [39]. In contrast, household financial constraints reflect the current economic situation of the household. The fact that education seems a stronger predictor of SEP inequalities in blood-pressure related risk factors as compared to household financial constraints is in line with another study using the same dataset but focusing on primary care [40]. Therefore, it is plausible to think that the educational opportunities and family background, reflected in attained education, have a more direct role in determining both lifestyles and subsequent biomarkers profiles, particularly blood pressure and CRP, than the current financial situation as captured by financial constraints. This implies differences are driven throughout the life course, and that current cross-sectional SEP measures are less informative as regards to inequalities in physiological risk biomarker levels.

Strong SEP inequalities were observed for blood pressure measures (SBP and DBP) analysed as continuous variables. These educational inequalities were slightly attenuated after adjustment for BMI and waist-hip ratio among women: for example the coefficient for DBP declined from − 1.53 (− 2.43 to − 0.63) to − 0.82 (− 1.69 to 0.05) on adjustment as shown in Table S2). However, this was not seen for men. These results are in line with evidence on the importance of obesity in SEP differentials among Russian women, but not among Russian men [41]. The fact that the other lifestyles (smoking, alcohol, fruit consumption and physical activity) contribute very little to the blood pressure profiles does not seem to be attributed to the categorization of the selected lifestyles, as different questions and categorizations were carefully explored. For example, the frequency of alcohol consumed or AUDIT score in a continuous scale did not explain more than the dichotomous AUDIT score ≥ 8 variable used in these analyses.

Alcohol has been established as a critically important risk factor in Russia, so the fact that it did not contribute to lowering the observed SEP inequalities in blood pressure levels deserves to be discussed. We need to acknowledge the important drops in harmful alcohol consumption and deaths from acute alcohol poisoning in Russia over the last 15 years [42]. These trends seem to be pointing towards important reductions in binge drinking. Previous studies on SEP in alcohol consumption in Russia suggest that while low SEP groups are more likely to engage in hazardous drinking they do not necessarily have an overall higher mean volume of alcohol consumed [31]. Nonetheless, our findings on the lack of contribution of alcohol to SEP inequalities in blood pressure is in line with recent research using US data that found alcohol not to be a main predictor of SEP differences in hypertension, cardiovascular disease or diabetes [43]. Therefore, other factors, which we were not able to fully adjust for, are likely to contribute to the remaining unexplained differences in blood pressure levels. For example, psychological factors and strategies to cope with stress, which have been often postulated to differences in health in Russia, or diet factors, which may mediate the association between socioeconomic status and risk factors [44, 45].

For HbA1C, the slightly higher levels observed among men from low SEP (see Table S2), particularly for education, seemed to be explained by the combination of established risk factors such as smoking or alcohol, and less by diet, physical activity, or obesity indicators (BMI plus waist-hip ratio). Considering the elevated share of undiagnosed diabetes in Russia [46], we could hypothesize about higher SEP inequalities on both self-reported and diagnosed diabetes. For cholesterol-based measures, SEP inequalities were only statistically significant for HDL among women and disappeared after adjusting for BMI and waist-hip ratio. This, however, does not necessarily mean that SEP inequalities in cholesterol do not exist, but they may require larger sample sizes to be identified.

Finally, CRP was the only risk biomarker in which clear financial constraints inequalities were found, although only in men. It could be that CRP is more strongly influenced by acute factors (e.g. psychological distress) than the other biomarkers analyzed. In addition, the clearly observed educational inequalities in CRP among women (model 1, OR: 0.66 (0.55-0.79)) seem to be largely explained by obesity-related measures (model 3, OR: 0.78 (0.64-0.93)), and are in line with previous studies suggesting large SEP inequalities in obesity among Russian women [41]. However, in both men and women inequalities existed in the odds of raised CRP levels even after adjusting for lifestyles. In this case, the OR is substantially attenuated after adjusting for smoking among men and for obesity-related measures among women. Thus, even after adjusting for lifestyles and medication educational inequalities persist in blood pressure among men and in CRP among women.

Overall, it seems that most of the potential explanations of the SEP inequalities were captured by BMI/waist-hip ratio, particularly among women. Again, the fact that other lifestyle factors seem to contribute very little to the observed SEP inequalities in risk biomarkers does not necessarily mean that inequalities do not exist, but that we were unable to find statistical evidence for them using cross-sectional data. This might indicate that the biomarkers analyzed could be more driven by chronic inequalities (as captured by education) and less by acute factors (as in part captured by current financial constraints).

In additional analyses, we also adjusted for other individual factors unrelated to lifestyle such as visits to the doctor, blood pressure knowledge, and medication use. For blood pressure, only medication use among women substantially reduced the SEP-coefficient, and therefore contributed to explaining SEP inequalities, while for the remaining risk factors adding these adjustments did not substantially change our estimates. The relationship between SEP and medication use is complex given there may be SEP gradients both in indications for use and access to medication, and associated adherence to treatment when medication is needed. As suggested by the results from this study when adding the multiple adjustments, our general conclusions would not change. We have accounted for it by adding risk factor specific medication as adjusted in Model 5. In doing so, the SEP coefficient remained generally similar for most of the analysed risk biomarkers. Nonetheless, we acknowledge that more detailed studies on the role of medication on these associations would be worthwhile.

### Evaluation of data and methods

This study has several limitations. First, we need to acknowledge that the sample comes from two Russian cities (Arkhangelsk and Novosibirsk) and are not based on nationally representative samples. The participation rate in KYH was 51% overall. This implies that we are subject to potential selection bias that may mean that we had lower power to detect true differences in the population if study participation was differential by SEP. Furthermore, we dichotomized the educational variable for increasing the statistical power, but we acknowledge that this implies a limitation in terms of assessing a full spectrum of educational inequalities. Our other key socioeconomic variable, self-reported financial constraints was previously used in other Russian studies [47] and accounts for a more subjective and updated dimension of socioeconomic status as compared to education. The KYH appears to be robust as its final sample had similar age and educational profiles as the Russian urban population [26], and the risk factor data is consistent with that previously observed, including smoking and blood pressure levels [48, 49].

This study used cross-sectional data of measured biomarkers levels and lifestyles (e.g. alcohol or tobacco use). It may be that some individuals have changed their lifestyle and improved their risk markers as a result of advice from medical doctors due to a CVD event which would attenuate the strength of the underlying associations. However, our sensitivity analysis for the sample free of CVD are in line with our main results, both in terms of the strength of the statistical evidence and importance of individual lifestyle factors (see Tables S1-S2), suggesting that this bias due to reverse causality may not be major.

A strength of this study is that we used actual individual risk factor measurements, as opposed to indicators of overall risk factor risk [25], only self-reported elevated prevalence of risk factors, as more commonly addressed [43]. One of the exceptions to this found more consistent association between SEP and biomarkers of CVD disease for women than for men using Australian data [50], which is in line with our findings.

## Conclusion

In this study, we have found important educational differences in in risk biomarkers, particularly in blood pressure and CRP. These inequalities generally diminished once adjusting for obesity-related measures for women, but not for men. Educational inequalities in physiological risk biomarkers could aid in explaining SEP inequalities in CVD health outcomes. Our findings provide evidence-based information on the need for tackling health inequalities in the Russian population, which may help as well to further contribute to CVD mortality decline. Public health policies aimed at prevention should acknowledge the observed gender differences in risk factor profiles and the role of lifestyle factors in explaining some of them, to reduce inequalities in risk factors and subsequent CVD health outcomes.

## Supplementary Information

**Additional file 1: Table S1A**. Odds ratio of high physiological CVD risk biomarkers with education and household financial constraints in the sample free of CVD, Know Your Heart, men. **Table S1B**. Odds ratio of high physiological CVD risk biomarkers with education and household financial constraints in the sample free of CVD, Know Your Heart, women. **Table S2A**. Associations of physiological CVD risk biomarkers levels with education and household financial constraints, Know Your Heart, men. **Table S2B**. Associations of physiological CVD risk biomarkers levels with education and household financial constraints, Know Your Heart, women.

## Abbreviations

AUDIT: Alcohol Use Disorders Identification Test
BMI: Body mass index
CVD: Cardiovascular disesase
CRP: C-reactive protein
DBP: Diastolic blood pressure
HbA1C: Glycated haemoglobin
HDL: High density lipoprotein
HMD: Human Mortality Database
IPCDR: International Project on Cardiovascular Disease in Russia
KYH: Know Your Heart Study
LDL: Low density lipoprotein
OR: Odds ratios
SAHR: Survey on Stress, Ageing and Health in Russia
SBP: Systolic blood pressur
SEP: Socioeconomic position
TCHOL: Total colesterol

## References

[1] Townsend N, Kazakiewicz D, Lucy Wright F, Timmis A, Huculeci R, Torbica A (2022). Epidemiology of cardiovascular disease in Europe. Nat Rev Cardiol..

[2] Timmis A, Townsend N, Gale CP, Torbica A, Lettino M, Petersen SE (2020). European Society of Cardiology: cardiovascular disease statistics 2019. Eur Heart J.

[3] Ezzati M, Obermeyer Z, Tzoulaki I, Mayosi BM, Elliott P, Leon DA (2015). Contributions of risk factors and medical care to cardiovascular mortality trends. Nat Rev Cardiol.

[4] Harper S, Lynch J, Smith GD (2011). Social determinants and the decline of cardiovascular diseases: understanding the links. Annu Rev Public Health.

[5] Steptoe A, Marmot M (2002). The role of psychobiological pathways in socio-economic inequalities in cardiovascular disease risk. Eur Heart J.

[6] Méjean C, Droomers M, van der Schouw YT, Sluijs I, Czernichow S, Grobbee DE (2013). The contribution of diet and lifestyle to socioeconomic inequalities in cardiovascular morbidity and mortality. Int J Cardiol.

[7] Stringhini S, Carmeli C, Jokela M, Avendaño M, Muennig P, Guida F (2017). Socioeconomic status and the 25 × 25 risk factors as determinants of premature mortality: a multicohort study and meta-analysis of 1·7 million men and women. Lancet.

[8] Trias-Llimós S, Pennells L, Tverdal A, Kudryavtsev AV, Malyutina S, Hopstock LA (2020). Quantifying the contribution of established risk factors to cardiovascular mortality differences between Russia and Norway. Sci Rep.

[9] Shkolnikov VM, Andreev EM, Tursun-Zade R, Leon DA (2019). Patterns in the relationship between life expectancy and gross domestic product in Russia in 2005–15: a cross-sectional analysis. Lancet Public Health.

[10] Kontsevaya A, Sabgaida T, Ivanova A, Leon DA, McKee M (2017). How has the management of acute coronary syndrome changed in the Russian Federation during the last 10 years?. Health Policy.

[11] Shkolnikov VM, Leon DA, Adamets S, Andreev E, Deev A (1998). Educational level and adult mortality in Russia: an analysis of routine data 1979 to 1994. Soc Sci Med.

[12] Shkolnikov VM (2006). The changing relation between education and life expectancy in central and eastern Europe in the 1990s. J Epidemiol Community Health.

[13] Krieger N, Williams DR, Moss NE (1997). Measuring social class in US public health research: concepts, methodologies, and guidelines. Annu Rev Public Health.

[14] Kunst AE (2009). Socioeconomic inequalities in health in central and Eastern Europe: synthesis of results of eight new studies. Int J Public Health.

[15] Di Girolamo C, Nusselder WJ, Bopp M, Brønnum-Hansen H, Costa G, Kovács K (2020). Progress in reducing inequalities in cardiovascular disease mortality in Europe. Heart..

[16] Mackenbach JP, Valverde JR, Artnik B, Bopp M, Brønnum-Hansen H, Deboosere P (2018). Trends in health inequalities in 27 European countries. Proc Natl Acad Sci.

[17] Murphy M, Bobak M, Nicholson A, Rose R, Marmot M (2006). The widening gap in mortality by educational level in the Russian Federation, 1980–2001. Am J Public Health.

[18] Malyutina S, Bobak M, Simonova G, Gafarov V, Nikitin Y, Marmot M (2004). Education, marital status, and total and cardiovascular mortality in Novosibirsk, Russia: a prospective cohort study. Ann Epidemiol.

[19] Todd MA, Shkolnikov VM, Goldman N (2016). Why are well-educated Muscovites more likely to survive? Understanding the biological pathways. Soc Sci Med.

[20] Dennis BH, Zhukovsky GS, Shestov DB, Davis CE, Deev A, Kim H (1993). The association of education with coronary heart disease mortality in the USSR lipid research clinics study. Int J Epidemiol.

[21] World Health Organization. Global status report on alcohol and health 2018. [Internet]. 2018. Available from: http://www.who.int/substance_abuse/publications/global_alcohol_report/en/. [cited 2019 Jun 10].

[22] Giovino GA, Mirza SA, Samet JM, Gupta PC, Jarvis MJ, Bhala N (2012). Tobacco use in 3 billion individuals from 16 countries: an analysis of nationally representative cross-sectional household surveys. Lancet.

[23] NCD Risk Factor Collaboration (2017). Worldwide trends in blood pressure from 1975 to 2015: a pooled analysis of 1479 population-based measurement studies with 19· 1 million participants. Lancet Lond Engl.

[24] Tillmann T, Pikhart H, Peasey A, Kubinova R, Pajak A, Tamosiunas A, et al. Psychosocial and socioeconomic determinants of cardiovascular mortality in Eastern Europe: a multicentre prospective cohort study. PLoS Med. 2017;14(12):e1002459. 10.1371/journal.pmed.1002459 PMC5718419 29211726

[25] Glei DA, Goldman N, Shkolnikov VM, Jdanov D, Shalnova S, Shkolnikova M (2013). To what extent do biomarkers account for the large social disparities in health in Moscow?. Soc Sci Med.

[26] Cook S, Malyutina S, Kudryavtsev A, et al. Know Your Heart: rationale, design and conduct of a cross-sectional study of cardiovascular structure, function, and risk factors in 4,500 men and women aged 35–69 years from two Russian cities, 2015–18. Wellcome Open Res. 2018;3:67. 10.12688/wellcomeopenres.14619.3. 10.12688/wellcomeopenres.14619.2 PMC6073094 30123849

[27] Imahori Y, Frost C, Mathiesen EB, Ryabikov A, Kudryavtsev AV, Malyutina S (2020). Effect of adiposity on differences in carotid plaque burden in studies conducted in Norway and Russia: a cross-sectional analysis of two populations at very different risk of cardiovascular mortality. BMJ Open.

[28] Levshin V, Slepchenko N. Determinants of smoking cessation and abstinence in a Russian smoking-cessation center. Tob Prev Cessat. 2017;3 Available from: https://www.ncbi.nlm.nih.gov/pmc/articles/PMC7232794/. [cited 2021 Dec 6]. 10.18332/tpc/76623 PMC7232794 32432198

[29] Babor TF, de la Fuente JR, Saunders J, Grant M (2001). The alcohol use disorders identification test: guidelines for use in primary care.

[30] Saunders JB, Aasland OG, Babor TF, De la Fuente JR, Grant M (1993). Development of the alcohol use disorders identification test (AUDIT): WHO collaborative project on early detection of persons with harmful alcohol consumption-II. Addiction..

[31] Cook S, De Stavola B, Saburova L, Kiryanov N, Vasiljev M, McCambridge J (2011). Socio-demographic predictors of dimensions of the AUDIT score in a population sample of working-age men in Izhevsk, Russia. Alcohol Alcohol.

[32] Cust AE, Smith BJ, Chau J, van der Ploeg HP, Friedenreich CM, Armstrong BK (2008). Validity and repeatability of the EPIC physical activity questionnaire: a validation study using accelerometers as an objective measure. Int J Behav Nutr Phys Act.

[33] Wareham NJ, Jakes RW, Rennie KL, Mitchell J, Hennings S, Day NE (2002). Validity and repeatability of the EPIC-Norfolk physical activity questionnaire. Int J Epidemiol.

[34] World Health Organization. Waist circumference and waist-hip ratio: report of a WHO expert consultation. Geneva; 2008. p. 2011.

[35] Toft U, Kristoffersen LH, Lau C, Borch-Johnsen K, Jørgensen T (2007). The dietary quality score: validation and association with cardiovascular risk factors: the Inter99 study. Eur J Clin Nutr.

[36] WHO Collaborating Centre for Drug Statistics Methodology [internet]. World health Organization; 2019. Available from: https://www.whocc.no/.

[37] Winkleby MA, Jatulis DE, Frank E, Fortmann SP (1992). Socioeconomic status and health: how education, income, and occupation contribute to risk factors for cardiovascular disease. Am J Public Health.

[38] Galobardes B, Shaw M, Lawlor DA, Lynch JW, Smith GD (2006). Indicators of socioeconomic position (part 1). J Epidemiol Community Health.

[39] Tillmann T, Vaucher J, Okbay A, Pikhart H, Peasey A, Kubinova R (2017). Education and coronary heart disease: mendelian randomisation study. BMJ.

[40] Petersen J, Kontsevaya A, McKee M, Richardson E, Cook S, Malyutina S (2020). Primary care use and cardiovascular disease risk in Russian 40–69 year olds: a cross-sectional study. J Epidemiol Community Health.

[41] Pikhart H, Bobak M, Malyutina S, Pajak A, Kubínová R, Marmot M (2007). Obesity and education in three countries of the central and Eastern Europe: the HAPIEE study. Cent Eur J Public Health.

[42] Danilova I, Shkolnikov VM, Andreev E, Leon DA (2020). The changing relation between alcohol and life expectancy in Russia in 1965–2017. Drug Alcohol Rev.

[43] Kino S, Kawachi I (2020). How much do preventive health behaviors explain education-and income-related inequalities in health? Results of Oaxaca–Blinder decomposition analysis. Ann Epidemiol.

[44] Boylan S, Lallukka T, Lahelma E, Pikhart H, Malyutina S, Pajak A (2011). Socio-economic circumstances and food habits in eastern, central and Western European populations. Public Health Nutr.

[45] Cockerham WC, Hinote BP, Abbott P (2006). Psychological distress, gender, and health lifestyles in Belarus, Kazakhstan, Russia, and Ukraine. Soc Sci Med.

[46] Iakunchykova O, Averina M, Wilsgaard T, Malyutina S, Kudryavtsev AV, Cook S, et al. What factors explain the much higher diabetes prevalence in Russia compared with Norway? Major sex differences in the contribution of adiposity. BMJ Open Diabetes Res Care. 2021;9(1):e002021. 10.1136/bmjdrc-2020-002021 PMC7934764 33664061

[47] Pridemore WA, Tomkins S, Eckhardt K, Kiryanov N, Saburova L (2010). A case–control analysis of socio-economic and marital status differentials in alcohol- and non-alcohol-related mortality among working-age Russian males. Eur J Pub Health.

[48] Balanova YA, Shalnova SA, Imaeva AE, Kapustina АV, Muromtseva GA, Evstifeeva SV (2019). Prevalence, Awareness, Treatment and Control of Hypertension in Russian Federation (Data of Observational ESSERF-2 Study). Rational Pharmacother Cardiol.

[49] Shkolnikov VM, Churilova E, Jdanov DA, Shalnova SA, Nilssen O, Kudryavtsev A (2020). Time trends in smoking in Russia in the light of recent tobacco control measures: synthesis of evidence from multiple sources. BMC Public Health.

[50] Kavanagh A, Bentley RJ, Turrell G, Shaw J, Dunstan D, Subramanian SV (2010). Socioeconomic position, gender, health behaviours and biomarkers of cardiovascular disease and diabetes. Soc Sci Med.

