# Supplementary material for: Socioeconomic inequalities in physiological risk biomarkers and the role of lifestyles among Russians aged 35-69 years
Source: Int J Equity Health. 2022 Apr 15;21:51. doi: 10.1186/s12939-022-01650-3 (PMC9013063; doi:10.1186/s12939-022-01650-3)
Supplement: Supplementary file 1 — Additional file 1: Table S1A. Odds ratio of high physiological CVD risk biomarkers with education and household financial constraints in the sample free of CVD, Know Your Heart, men. Table S1B. Odds ratio of high physiological CVD risk biomarkers with education and household financial constraints in the sample free of CVD, Know Your Heart, women. Table S2A. Associations of physiological CVD risk biomarkers levels with education and household financial constraints, Know Your Heart, men. Table S2B. Associations of physiological CVD risk biomarkers levels with education and household financial constraints, Know Your Heart, women. [file 12939_2022_1650_MOESM1_ESM.docx]

**Supplementary material**

**Table S1A.** Odds ratio of high physiological CVD risk biomarkers with education and household financial constraints in the sample free of CVD, Know Your Heart, men

| **Men** |  | Model 1: Age + city | Model 2: Model 1 + smoking + alcohol (AUDIT8) + fruit + stairs | Model 3: Model 1 + BMI + waist-hip | Model 4: Models 2 + 3 | Model 5: Model 4 + medication |
| --- | --- | --- | --- | --- | --- | --- |
| Education (ref. Low) | SBP | **0.768 (0.615, 0.958)** | **0.752 (0.596, 0.948)** | **0.779 (0.623, 0.974)** | **0.766 (0.606, 0.968)** | **0.755 (0.597, 0.955)** |
|  | DBP | **0.800 (0.640, 0.999)** | **0.772 (0.611, 0.976)** | 0.810 (0.646, 1.015) | 0.792 (0.624, 1.005) | 0.788 (0.621, 1.000) |
|  | HDL | 0.801 (0.605, 1.060) | 0.875 (0.651, 1.176) | 0.831 (0.624, 1.106) | 0.933 (0.690, 1.262) | 0.924 (0.682, 1.250) |
|  | LDL | 0.966 (0.782, 1.194) | 0.938 (0.751, 1.170) | 0.997 (0.803, 1.237) | 0.979 (0.781, 1.227) | 0.992 (0.791, 1.245) |
|  | TCHOL | 0.922 (0.716, 1.187) | 0.892 (0.684, 1.163) | 0.941 (0.729, 1.215) | 0.909 (0.695, 1.188) | 0.913 (0.698, 1.195) |
|  | Triglycerides | 0.842 (0.641, 1.107) | 0.888 (0.666, 1.182) | 0.881 (0.663, 1.172) | 0.959 (0.711, 1.293) | 0.947 (0.702, 1.277) |
|  | HbA1C | 0.984 (0.590, 1.641) | 1.091 (0.637, 1.869) | 1.021 (0.605, 1.721) | 1.253 (0.716, 2.191) | 1.170 (0.664, 2.061) |
|  | CRP | 0.853 (0.671, 1.083) | 1.044 (0.808, 1.349) | 0.871 (0.683, 1.110) | 1.088 (0.838, 1.413) | 1.076 (0.828, 1.398) |
| Financial constraints (ref. Low) | SBP | 0.862 (0.687, 1.083) | 0.877 (0.693, 1.111) | 0.887 (0.705, 1.117) | 0.903 (0.711, 1.147) | 0.907 (0.714, 1.153) |
|  | DBP | 0.995 (0.792, 1.248) | 1.018 (0.803, 1.291) | 1.011 (0.801, 1.275) | 1.042 (0.817, 1.329) | 1.044 (0.819, 1.332) |
|  | HDL | 0.944 (0.710, 1.256) | 0.985 (0.733, 1.325) | 0.966 (0.721, 1.295) | 1.036 (0.763, 1.407) | 1.042 (0.767, 1.415) |
|  | LDL | 1.008 (0.810, 1.253) | 1.000 (0.799, 1.253) | 0.998 (0.798, 1.247) | 0.992 (0.788, 1.250) | 0.986 (0.782, 1.243) |
|  | TCHOL | 1.002 (0.771, 1.301) | 1.007 (0.767, 1.322) | 0.988 (0.758, 1.288) | 0.985 (0.746, 1.299) | 0.983 (0.745, 1.297) |
|  | Triglycerides | 0.788 (0.592, 1.051) | 0.766 (0.568, 1.034) | 0.814 (0.603, 1.099) | 0.826 (0.604, 1.129) | 0.830 (0.606, 1.135) |
|  | HbA1C | 1.075 (0.633, 1.823) | 1.161 (0.668, 2.017) | 1.143 (0.663, 1.971) | 1.317 (0.738, 2.350) | 1.325 (0.742, 2.367) |
|  | CRP | **0.696 (0.541, 0.894)** | 0.804 (0.618, 1.046) | **0.708 (0.549, 0.914)** | 0.831 (0.635, 1.087) | 0.834 (0.637, 1.091) |

* SBP stands for systolic blood pressure, DBP for diastolic blood pressure, HDL for high density lipoprotein, LDL for low density lipoprotein, TCHOL for total cholesterol in blood, HbA1C for glycated haemoglobin and CRP for C-reactive protein.

**Table S1B.** Odds ratio of high physiological CVD risk biomarkers with education and household financial constraints in the sample free of CVD, Know Your Heart, women

| **Women** | | Model 1: Age + city | Model 2: Model 1 + smoking + alcohol (AUDIT8) + fruit + stairs | Model 3: Model 1 + BMI + waist-hip | Model 4: Models 2 + 3 | Model 5: Model 4 + medication |
| --- | --- | --- | --- | --- | --- | --- |
| Education (ref. Low) | SBP | **0.714 (0.570, 0.894)** | **0.756 (0.600, 0.953)** | **0.776 (0.617, 0.976)** | 0.823 (0.650, 1.041) | 0.856 (0.675, 1.086) |
|  | DBP | **0.685 (0.539, 0.870)** | **0.751 (0.586, 0.961)** | **0.771 (0.603, 0.985)** | 0.836 (0.650, 1.077) | 0.860 (0.667, 1.110) |
|  | HDL | **0.477 (0.301, 0.758)** | 0.583 (0.362, 0.939) | 0.586 (0.366, 0.940) | 0.707 (0.434, 1.151) | 0.710 (0.436, 1.156) |
|  | LDL | 1.019 (0.846, 1.228) | 1.025 (0.846, 1.241) | 1.083 (0.896, 1.309) | 1.082 (0.891, 1.313) | 1.070 (0.881, 1.300) |
|  | TCHOL | 1.271 (1.031, 1.567) | 1.292 (1.043, 1.601) | 1.305 (1.056, 1.612) | 1.318 (1.061, 1.636) | 1.305 (1.050, 1.621) |
|  | Triglycerides | 0.828 (0.631, 1.087) | 0.866 (0.655, 1.145) | 0.990 (0.746, 1.312) | 1.018 (0.762, 1.359) | 1.042 (0.780, 1.393) |
|  | HbA1C | 0.850 (0.554, 1.304) | 0.838 (0.541, 1.298) | 1.057 (0.677, 1.650) | 1.010 (0.639, 1.597) | 1.059 (0.669, 1.678) |
|  | CRP | **0.655 (0.538, 0.798)** | **0.682 (0.557, 0.835)** | **0.787 (0.635, 0.975)** | 0.809 (0.649, 1.007) | 0.827 (0.664, 1.031) |
| Financial constraints (ref. Low) | SBP | 0.847 (0.661, 1.084) | 0.892 (0.692, 1.149) | 0.879 (0.684, 1.130) | 0.926 (0.716, 1.197) | 0.952 (0.734, 1.234) |
|  | DBP | 0.883 (0.683, 1.141) | 0.942 (0.724, 1.225) | 0.928 (0.715, 1.206) | 0.981 (0.750, 1.283) | 1.002 (0.765, 1.312) |
|  | HDL | 0.708 (0.433, 1.157) | 0.843 (0.508, 1.399) | 0.766 (0.466, 1.261) | 0.919 (0.549, 1.539) | 0.925 (0.552, 1.549) |
|  | LDL | 1.045 (0.854, 1.278) | 1.057 (0.861, 1.298) | 1.062 (0.866, 1.301) | 1.068 (0.869, 1.314) | 1.061 (0.863, 1.305) |
|  | TCHOL | 1.130 (0.900, 1.418) | 1.132 (0.898, 1.427) | 1.137 (0.906, 1.428) | 1.134 (0.900, 1.430) | 1.126 (0.892, 1.420) |
|  | Triglycerides | 1.051 (0.787, 1.405) | 1.087 (0.808, 1.463) | 1.133 (0.841, 1.528) | 1.156 (0.852, 1.570) | 1.177 (0.866, 1.599) |
|  | HbA1C | 1.005 (0.631, 1.602) | 1.032 (0.640, 1.665) | 1.075 (0.666, 1.735) | 1.106 (0.674, 1.814) | 1.108 (0.674, 1.824) |
|  | CRP | 0.884 (0.715, 1.093) | 0.926 (0.746, 1.150) | 0.944 (0.750, 1.188) | 0.986 (0.780, 1.247) | 1.003 (0.793, 1.269) |

* SBP stands for systolic blood pressure, DBP for diastolic blood pressure, HDL for high density lipoprotein, LDL for low density lipoprotein, TCHOL for total cholesterol in blood, HbA1C for glycated haemoglobin and CRP for C-reactive protein.

**Table S2A.** Associations of physiological CVD risk biomarkers levels with education and household financial constraints, Know Your Heart, men

| **Men** |  | Model 1: Age + city | Model 2: Model 1 + smoking + alcohol (AUDIT8) + fruit + stairs | Model 3: Model 1 + BMI + waist-hip | Model 4: Models 2 + 3 | Model 5: Model 4 + medication |
| --- | --- | --- | --- | --- | --- | --- |
| Education (ref. Low) | SBP (mmHg) | **-2.383 (-4.198, -0.567)** | **-2.555 (-4.438, -0.673)** | **-2.297 (-4.108, -0.487)** | **-2.435 (-4.314, -0.556)** | **-2.463 (-4.341, -0.585)** |
|  | DBP (mmHg) | **-1.631 (-2.703, -0.559)** | **-1.818 (-2.917, -0.718)** | **-1.530 (-2.584, -0.476)** | **-1.669 (-2.752, -0.586)** | **-1.675 (-2.758, -0.591)** |
|  | HDL (mmol/L) | 0.005 (-0.026, 0.036) | -0.004 (-0.035, 0.028) | 0.007 (-0.023, 0.036) | -0.007 (-0.036, 0.023) | -0.007 (-0.036, 0.023) |
|  | LDL (mmol/L) | 0.001 (-0.082, 0.085) | -0.015 (-0.102, 0.072) | 0.001 (-0.082, 0.084) | -0.008 (-0.094, 0.079) | -0.009 (-0.091, 0.074) |
|  | TCHOL (mmol/L) | 0.007 (-0.097, 0.111) | -0.011 (-0.118, 0.096) | 0.006 (-0.097, 0.110) | -0.004 (-0.111, 0.103) | -0.005 (-0.108, 0.097) |
|  | log Triglycerides (mmol/L) | 0.020 (-0.036, 0.076) | 0.023 (-0.034, 0.081) | 0.017 (-0.034, 0.069) | 0.032 (-0.022, 0.085) | 0.032 (-0.022, 0.085) |
|  | HbA1C (%) | **-0.095 (-0.174, -0.016)** | -0.080 (-0.162, 0.002) | **-0.096 (-0.173, -0.019)** | -0.071 (-0.151, 0.008) | -0.073 (-0.149, 0.002) |
|  | CRP (mg/L) | **-0.928 (-1.714, -0.143)** | -0.507 (-1.323, 0.309) | **-0.938 (-1.722, -0.154)** | -0.472 (-1.285, 0.340) | -0.472 (-1.285, 0.341) |
| Financial constraints (ref. Low) | SBP (mmHg) | -1.119 (-3.018, 0.779) | -1.014 (-2.962, 0.935) | -1.057 (-2.956, 0.843) | -0.935 (-2.885, 1.015) | -0.841 (-2.792, 1.110) |
|  | DBP (mmHg) | -0.318 (-1.443, 0.808) | -0.201 (-1.344, 0.941) | -0.239 (-1.349, 0.872) | -0.098 (-1.226, 1.030) | -0.082 (-1.212, 1.048) |
|  | HDL (mmol/L) | 0.003 (-0.030, 0.035) | 0.000 (-0.032, 0.032) | 0.008 (-0.023, 0.039) | 0.002 (-0.029, 0.033) | 0.000 (-0.031, 0.030) |
|  | LDL (mmol/L) | 0.053 (-0.035, 0.141) | 0.043 (-0.047, 0.133) | 0.042 (-0.045, 0.129) | 0.034 (-0.055, 0.124) | -0.001 (-0.086, 0.085) |
|  | TCHOL (mmol/L) | 0.049 (-0.060, 0.158) | 0.038 (-0.073, 0.148) | 0.038 (-0.071, 0.147) | 0.028 (-0.082, 0.139) | -0.012 (-0.119, 0.094) |
|  | log Triglycerides (mmol/L) | -0.015 (-0.074, 0.043) | -0.024 (-0.083, 0.036) | -0.024 (-0.079, 0.030) | -0.026 (-0.081, 0.030) | -0.028 (-0.084, 0.027) |
|  | HbA1C (%) | -0.039 (-0.122, 0.043) | -0.034 (-0.118, 0.050) | -0.045 (-0.125, 0.035) | -0.032 (-0.114, 0.050) | -0.029 (-0.106, 0.049) |
|  | CRP (mg/L) | -0.804 (-1.620, 0.011) | -0.472 (-1.307, 0.362) | -0.769 (-1.585, 0.048) | -0.414 (-1.248, 0.420) | -0.414 (-1.248, 0.421) |

* SBP stands for systolic blood pressure, DBP for diastolic blood pressure, HDL for high density lipoprotein, LDL for low density lipoprotein, TCHOL for total cholesterol in blood, HbA1C for glycated haemoglobin and CRP for C-reactive protein.

**Table S2B.** Associations of physiological CVD risk biomarkers levels with education and household financial constraints, Know Your Heart, women

| **Women** |  | Model 1: Age + city | Model 2: Model 1 + smoking + alcohol (AUDIT8) + fruit + stairs | Model 3: Model 1 + BMI + waist-hip | Model 4: Models 2 + 3 | Model 5: Model 4 + medication |
| --- | --- | --- | --- | --- | --- | --- |
| Education (ref. Low) | SBP (mmHg) | **-3.113 (-4.606, -1.619)** | **-3.009 (-4.539, -1.479)** | **-2.158 (-3.625, -0.691)** | **-2.110 (-3.610, -0.611)** | **-1.743 (-3.224, -0.262)** |
|  | DBP (mmHg) | **-1.530 (-2.425, -0.634)** | **-1.232 (-2.146, -0.318)** | -0.818 (-1.686, 0.049) | -0.583 (-1.468, 0.302) | -0.425 (-1.305, 0.455) |
|  | HDL (mmol/L) | **0.044 ( 0.016, 0.073)** | **0.038 ( 0.009, 0.067)** | 0.011 (-0.016, 0.038) | 0.008 (-0.020, 0.035) | 0.009 (-0.019, 0.036) |
|  | LDL (mmol/L) | -0.005 (-0.078, 0.068) | -0.002 (-0.077, 0.072) | 0.011 (-0.063, 0.084) | 0.012 (-0.063, 0.087) | 0.026 (-0.047, 0.098) |
|  | TCHOL (mmol/L) | 0.014 (-0.076, 0.105) | 0.013 (-0.080, 0.105) | 0.020 (-0.071, 0.111) | 0.018 (-0.076, 0.111) | 0.034 (-0.057, 0.124) |
|  | log Triglycerides (mmol/L) | -0.050 (-0.092, -0.008) | -0.039 (-0.082, 0.004) | 0.000 (-0.040, 0.039) | 0.005 (-0.035, 0.045) | 0.005 (-0.035, 0.045) |
|  | HbA1C (%) | **0.006 (-0.056, 0.068)** | 0.007 (-0.057, 0.070) | 0.049 (-0.012, 0.110) | 0.044 (-0.019, 0.106) | 0.042 (-0.017, 0.101) |
|  | CRP (mg/L) | **-0.512 (-0.930, -0.094)** | **-0.443 (-0.872, -0.014)** | -0.148 (-0.556, 0.260) | -0.105 (-0.523, 0.313) | -0.105 (-0.523, 0.312) |
| Financial constraints (ref. Low) | SBP (mmHg) | -0.937 (-2.586, 0.711) | -0.708 (-2.380, 0.965) | -0.524 (-2.128, 1.080) | -0.341 (-1.968, 1.286) | -0.037 (-1.642, 1.567) |
|  | DBP (mmHg) | -0.715 (-1.702, 0.273) | -0.525 (-1.523, 0.474) | -0.443 (-1.390, 0.504) | -0.292 (-1.251, 0.667) | -0.161 (-1.114, 0.792) |
|  | HDL (mmol/L) | 0.020 (-0.011, 0.052) | 0.015 (-0.017, 0.047) | 0.008 (-0.021, 0.037) | 0.004 (-0.025, 0.034) | 0.004 (-0.026, 0.033) |
|  | LDL (mmol/L) | -0.018 (-0.098, 0.062) | -0.015 (-0.096, 0.066) | -0.014 (-0.094, 0.066) | -0.012 (-0.093, 0.069) | -0.024 (-0.103, 0.055) |
|  | TCHOL (mmol/L) | 0.002 (-0.097, 0.101) | 0.002 (-0.098, 0.103) | 0.002 (-0.097, 0.102) | 0.002 (-0.099, 0.102) | -0.013 (-0.111, 0.085) |
|  | log Triglycerides (mmol/L) | 0.006 (-0.040, 0.053) | 0.013 (-0.034, 0.060) | 0.025 (-0.018, 0.068) | 0.029 (-0.015, 0.072) | 0.028 (-0.015, 0.072) |
|  | HbA1C (%) | 0.020 (-0.048, 0.088) | 0.018 (-0.051, 0.087) | 0.037 (-0.030, 0.104) | 0.031 (-0.036, 0.099) | 0.038 (-0.026, 0.102) |
|  | CRP (mg/L) | -0.452 (-0.915, 0.011) | -0.427 (-0.897, 0.043) | -0.327 (-0.775, 0.121) | -0.323 (-0.777, 0.132) | -0.318 (-0.772, 0.137) |

* SBP stands for systolic blood pressure, DBP for diastolic blood pressure, HDL for high density lipoprotein, LDL for low density lipoprotein, TCHOL for total cholesterol in blood, HbA1C for glycated haemoglobin and CRP for C-reactive protein.
